# Intratumoral Th2 predisposition combines with an increased Th1 functional phenotype in clinical response to intravesical BCG in bladder cancer

**DOI:** 10.1007/s00262-016-1945-z

**Published:** 2016-12-22

**Authors:** Renate Pichler, Georg Gruenbacher, Zoran Culig, Andrea Brunner, Dietmar Fuchs, Josef Fritz, Hubert Gander, Andrea Rahm, Martin Thurnher

**Affiliations:** 1 Department of Urology, Research Group of Urologic Oncology, Medical University of Innsbruck, Anichstrasse 35, 6020 Innsbruck, Austria; 2 Immunotherapy Research Unit, Medical University of Innsbruck, Innsbruck, Austria; 3 Division of Experimental Urology, Medical University of Innsbruck, Innsbruck, Austria; 4 Division of General Pathology, Department of Pathology, Medical University of Innsbruck, Innsbruck, Austria; 5 Division of Biological Chemistry, Biocenter, Medical University of Innsbruck, Innsbruck, Austria; 6 Department of Medical Statistics, Informatics and Health Economics, Medical University of Innsbruck, Innsbruck, Austria

**Keywords:** BCG, Bladder cancer, T cells, Neopterin, GATA3, T-bet

## Abstract

Th1-type immunity is considered to be required for efficient response to BCG in bladder cancer, although Th2 predisposition of BCG responders has recently been reported. The aim was to evaluate the relationship of Th1 and Th2 components in 23 patients undergoing BCG treatment. Peripheral blood, serum and urine samples were prospectively collected at baseline, during and after BCG. Th1 (neopterin, tryptophan, kynurenine, kynurenine-to-tryptophan ratio (KTR), IL-12, IFN-γ, soluble TNF-R75 and IL-2Rα) and Th2 (IL-4, IL-10) biomarkers as well as CD4 expression in T helper (Th), effector and regulatory T cells were determined. Local immune cell subsets were measured on formalin-fixed, paraffin-embedded cancer tissue by immunohistochemistry to examine expression of transcription factors that control Th1 (T-bet) and Th2-type (GATA3) immunity. We confirmed a Th2 predisposition with a mean GATA3/T-bet ratio of 5.51. BCG responders showed significantly higher levels of urinary (*p* = 0.003) and serum neopterin (*p* = 0.012), kynurenine (*p* = 0.015), KTR (*p* = 0.005), IFN-γ (*p* = 0.005) and IL-12 (*p* = 0.003) during therapy, whereas levels of IL-10 decreased significantly (*p* < 0.001) compared to non-responders. GATA3/T-bet ratio correlated positively with serum neopterin (*p* = 0.008), IFN-γ (*p* = 0.013) and KTR (*p* = 0.018) after the first BCG instillation. We observed a significant increase in CD4 expression in the Th cell population (*p* < 0.05), with only a modest tendency toward higher frequency in responders compared to non-responders (*p* = 0.303). The combined assessment of GATA3/T-bet ratio, neopterin and KTR may be a useful biomarker in predicting BCG response. Th2-promoting factors such as GATA3 may trigger Th1-type immune responses and thus contribute to the BCG success.

**Electronic supplementary material:**

The online version of this article (doi:10.1007/s00262-016-1945-z) contains supplementary material, which is available to authorized users.

## Introduction

After decades of skepticism, immunotherapy is poised to become a mainstay of cancer treatment [1]. Currently, the most promising approach in activating therapeutic antitumor immunity is the blockade of immune checkpoints [2] as well as the use of genetically engineered T cells with chimeric antigen receptor (CAR) directed against tumor-associated antigens [3]. Treatment with Bacillus Calmette–Guérin (BCG) belongs to the most successful cancer immunotherapies, and in high-risk, non-muscle-invasive bladder cancer (NMIBC), it is the standard adjuvant treatment according to the European Association of Urology (EAU) guidelines [4]. Forty years after its introduction [5], the exact immune mechanism of BCG-induced antitumor activity is still not fully understood. Following BCG instillations, intravesical BCG–fibronectin complexes are internalized at the tumor resection site [6]. Antigen-presenting cells in the urothelium can phagocytose BCG and present BCG-derived antigens to CD4^+^ T cells. *In vitro* work by Brandau et al. has demonstrated that BCG activates natural killer (NK) cells in a monocyte-dependent manner [7]. It is well established that innate lymphocytes including NK cells not only participate in the early innate response but also promote and shape the subsequent adaptive response by triggering dendritic cell maturation [8] and are therefore essential for effective BCG immunotherapy [9, 10]. Different cytokines such as interleukin (IL)-1, IL-2, IL-6, IL-7, IL-8, IL-10, IL-12, tumor necrosis factor-(TNF)-α and interferon (IFN)-γ are released and can be detected in patients treated with BCG [11–13]. Thus, BCG can induce the production of both Th1-type and Th2-type cytokines. This fact was confirmed in vitro showing that BCG stimulates cultured murine dendritic cells, which are able to induce both IL-12 and IL-10, resulting in a mixed, nontargeted Th1 and Th2 immune response [14]. However, a predominant Th1 cell-mediated immunity with an enhanced recognition of cancer cells through infiltrating effector cells into the bladder wall is required for subsequent BCG response [15]. IL-12- or IFN-γ-depleted animals were BCG-resistant with a poor cancer-specific survival [16], whereas therapeutic strategies administering BCG along with Th1 cytokines and concurrent blocking of Th2 cells may enhance BCG-induced IFN-γ production and BCG vaccine efficacy [17–20]. Moreover, significant increases in urine concentrations of Th1-type cytokines during treatment were noticed in BCG responders [21, 22].

IFN-γ is an important stimulus for the enzyme GTP cyclohydrolase (GCH-I) in human monocyte-derived macrophages and dendritic cells, which induces neopterin production reflecting cellular immune activation [23, 24]. In parallel, IFN-γ activates the enzyme indoleamine 2,3-dioxygenase (IDO1), which converts tryptophan to kynurenine resulting in increased tryptophan breakdown, and elevated kynurenine-to-tryptophan ratio (KTR), [23]. Therefore, neopterin production and tryptophan breakdown are surrogate markers of IFN-γ production and thus of an ongoing Th1-type immune response. Currently, only a letter to the editor reported monitoring of neopterin in bladder cancer patients during intravesical BCG therapy [25]. Moreover, intravesical instillations of autologous IFN-γ-activated macrophages resulted in an increase in urinary neopterin [26].

It is well known that differentiation of type 1 and type 2 Th cells [27] as well as innate lymphoid cells [28] is controlled by the transcription factors T-bet and GATA3. Interestingly, a genome-wide analysis has revealed that T-bet is sufficient to induce GATA3 binding at Th1 specific sites, indicating its direct influence and responsibility for the redistribution of GATA3 in Th1 cells [29].

Recently, we confirmed a Th2 predisposition (GATA3>T-bet) of tumor-infiltrating immune cells in high-risk NMIBC patients with response to BCG [30]. The aim of the present follow-up study was to examine the relation between such a Th2 predisposition and the actual functional phenotype during treatment as a potential biomarker of BCG response.

## Materials and methods

### Patients

**Fig. 1:**
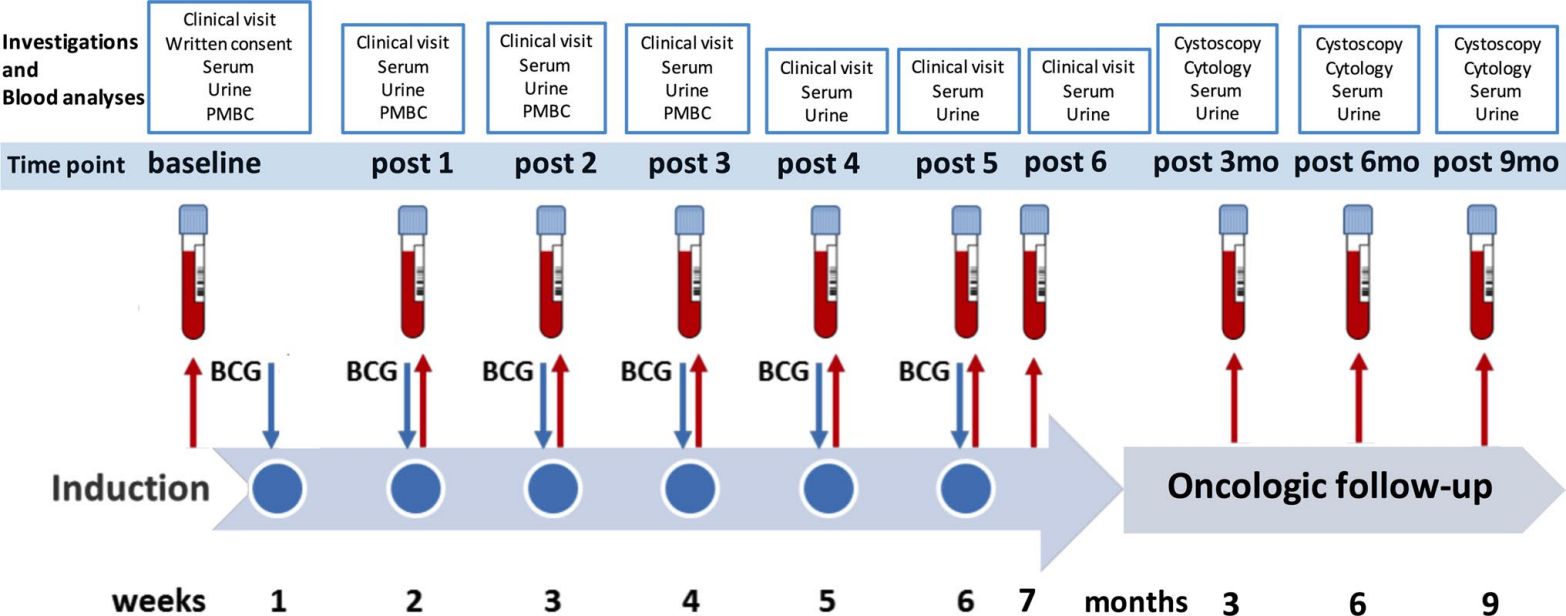
Prospective study design showing the planned investigations and blood analyses at each visit (baseline, during and after BCG therapy). *PBMC* peripheral blood mononuclear cells, after (post), *BCG* Bacillus Calmette–Guérin

This prospective study was approved by the local ethical committee of the Medical University of Innsbruck (study number AN2014-0121; 336/4.3), and written informed consent was obtained before study inclusion. All patients with primary NMIBC who had undergone transurethral resection of the bladder (TURB) from March 2014 to April 2015 with consecutive intravesical BCG immunotherapy were enrolled in this study. A second TURB was performed in all patients (except primary, isolated carcinoma in situ) before starting BCG induction and maintenance at our outpatient department. Each instillation contained 2 × 10^8^–3 × 10^9^ viable units from live attenuated BCG bacteria strain seed RIVM derived from seed 1173-P2 (BCG Medac, Wedel, Germany). Follow-up included cystoscopy and urinary cytology (voided urine and bladder washing) 3-monthly, and upper urinary tract imaging (CT urography or intravenous urography) once a year and in case of cancer recurrence [4]. A muscle-invasive bladder cancer detected during follow-up or a high-grade recurrence after completion of therapy was defined as BCG failure. BCG responders were defined as patients without any recurrence or evidence of disease based on follow-up cystoscopy and urinary cytology. A flowchart of the study design is shown in Fig. 1.

### Sample collection, preparation and cryopreservation

Heparinized whole blood, serum and urinary samples were collected at 10 different time points: baseline (before first BCG instillation), during BCG induction (7 days after each of the six BCG instillations) as well as during follow-up (at 3, 6 and 9 months). Peripheral blood mononuclear cells (PBMCs) were prepared from heparinized whole blood by Ficoll density centrifugation, and aliquots (5 × 10^6^ cells) were cryopreserved in liquid nitrogen. Serum samples were obtained under standard conditions, clotted at 4–8 °C and then centrifuged at 3200 rpm for 6 min. Aliquots of 1.8 ml were stored at −80 °C.

### Measurement of Th1- and Th2-related inflammatory metabolites, cytokines and soluble cytokine receptors

Concentrations of serum neopterin were determined by a commercially available ELISA according to the manufacturer’s instructions (BRAHMS Diagnostica, Hennigsdorf, Germany). Determination of urinary neopterin was performed using reversed phase (e.g., C18) HPLC with Sörensen phosphate buffer (e.g., 0.015 M, pH = 6.4, flow rate = 1.0 ml/min). Urinary neopterin was detected by measurement of its natural fluorescence (excitation wavelength 353 nm, emission wavelength 438 nm). To exclude an inflammation as a cause of neopterin increase, serum levels of C-reactive protein (CRP) were measured at the same time points. Creatinine concentrations were measured in parallel in the same chromatographic run by detection of its UV absorption at 235 nm. Measurement of kynurenine and tryptophan levels was taken by HPLC as described previously [31, 32], using an UV-spectrometric detector (SPD-6A, Shimadzu), a fluorescence detector (model 360, Varian ProStar), a Varian ProStar HPLC system with a solvent delivery module 210 and an autosampler (model 400, Varian ProStar). Kynurenine and tryptophan standards were purchased from Sigma (Steinheim, Germany). KTR was calculated and expressed as μmol/mmol [31]. Levels of urinary sIL-2Rα, sTNF-R75 and serum IFN-γ, IL-12 and IL-10 were determined by ELISA (R&D Systems Europe, Ltd., Abingdon, UK) following the manufacturer’s protocol.

### Flow cytometric analyses (FACS)

Subset analyses of freshly isolated non-activated PBMCs were performed to determine dynamic changes of the CD4 expression in Th cells, effector T cells (T_eff_) and regulatory T cells (T_reg_). CD3^+^ T cells were selectively analyzed for the expression of CD4 and CD25. T_eff_ (CD4^+^CD25^high^CD161^+^) cells were identified by additional staining of CD161, a pan-cancer prognostic gene (http://precog.stanford.edu). CD161 defines T cells with a conserved transcriptional signature and the ability to perform T cell receptor-independent, innate-like IFN-γ production in response to IL-12 and IL-18 [33]. This is in line with our own recent work demonstrating that CD4^+^CD161^+^ T cells produce IFN-γ in response to dendritic cell stimulation [34]. In contrast, T_reg_ cells were identified by high-level expression of CD25 and additional expression of CD39 (T_reg_ cells: CD4^+^CD25^+^CD39^+^ cells), [35]. The following fluorophore-conjugated monoclonal antibodies were used for phenotyping of PBMCs: anti-CD3 (UCHT-1-BV510; BD Biosciences), anti-CD4 (MT466-FITC; Miltenyi Biotec), anti-CD25 (2A3-PE; BD Biosciences), anti-CD39 (TU66-BV421; BD Biosciences) and anti-CD161 (HP-3G10-PerCP-Cy5.5; eBioscience). Cells were stained in PBS containing 0.5% FCS and 50 μg/ml human IgG (Octapharma) to block Fc γ receptors. After incubation for 30 min at 4 °C, cells were washed two times, resuspended and analyzed in a FACSCanto II flow cytometer using the FACS Diva 6.1.2 as well as FlowJo V7.2.5 software (BD Biosciences). Fixable viability dye eFluor 780 was used to label dead cells, and eFluor 780-negative cells were gated for further analysis (live cell selection), Supplementary Figure. 1.

### Immunohistochemistry (IHC)

To evaluate the predisposition for a Th1/Th2 tumor microenvironment prior to BCG, the density of Th1 and Th2 cells in tumor-infiltrating immune cells was measured on formalin-fixed, paraffin-embedded tissue sections of bladder cancer (in the lamina propria without invasion, at the invasive front, within the neoplastic urothelium and within the papillary stroma) by immunohistochemistry, using a T-bet antibody (monoclonal rabbit antihuman T-bet, MRQ-46, prediluted, Roche) and a GATA3 antibody (monoclonal mouse antihuman GATA3, L50-823, prediluted, Roche), which we had already validated in a recently work [30]. IHC staining was performed using an automated immunostainer (BenchMark ULTRA, Ventana Medical Systems, Tucson, US) according to the manufacturer’s protocol. We manually counted the total density of positive cells for each subset in up to 5 high-power fields (HPF) in each region, using the same field of view in consecutive slides. Microscope images were taken with an Olympus BX50 microscope (40x magnification) equipped with the ProgResC10plus camera (Jenoptik, Jena, Germany). IHC evaluation was performed by an experienced uropathologist.

### Statistics

Descriptive statistics (absolute and relative frequencies for qualitative data; mean and standard deviation (SD) for quantitative data) are given for all variables of interest. Bias-corrected and accelerated 95% bootstrapping confidence intervals (CIs) based on 2000 iterations were calculated for the means of all biomarkers at all time points. Changes over time in parameters were assessed using the Skillings–Mack test designed for block designs with missing observations [36]. Differences between BCG responders and non-responders were evaluated using a mixed model for repeated measurements with BCG response, time point and the interaction term as fixed effects and an AR(1) model for the within-subject covariance structure. Differences between single time points were evaluated using Mann–Whitney *U* tests. Correlations between parameters were assessed with Spearman’s *ρ* correlation coefficient (*r*_s_). The predictive power of biomarker levels on BCG response was evaluated by plotting receiver operating characteristic (ROC) curves and calculating the area under the curve (AUC). Expression levels were compared between tumor areas with the Friedman test and Wilcoxon signed-rank tests for pairwise comparisons. A significance level of *α* = 0.05 (two-tailed) was applied for all p values. Statistical analyses were performed using SPSS version 22 software (IBM Corp., Armonk, NY). Graphics were produced with Microsoft Excel and GraphPad PrismTM6 (GraphPad Software Inc., La Jolla, CA).

## Results

### Patients’ characteristics

A total of 23 (20 male and 3 female) patients with a mean (range) age of 71.3 (55–80) years were included. Histopathological evaluation confirmed a primary, high-risk NMIBC according to the European Organization for Research and Treatment of Cancer (EORTC) scoring system and risk tables [37]. Three patients showed low-grade carcinomas, while 20 (87.0%) of 23 patients had a high-grade cancer according to the WHO 2004 classification. Second TURB was performed in 19 (82.6%) patients, with tumor-free status in 12 (63.1%) patients, respectively. Descriptive and histopathological characteristics are shown in Supplementary Table 1. During a mean (range) follow-up of 16.2 (13–25) months, 19 (82.6%) patients were classified as BCG responders, while four (17.4%) patients showed BCG failure (multifocal, pT1 high-grade urothelial carcinoma and concurrent CIS) after a mean (range) follow-up of 7.5 (4–10) months. All those patients with BCG failure subsequently underwent radical cystectomy.

### Th2 predisposition of tumor-infiltrating immune cells prior to BCG therapy

**Fig. 2:**
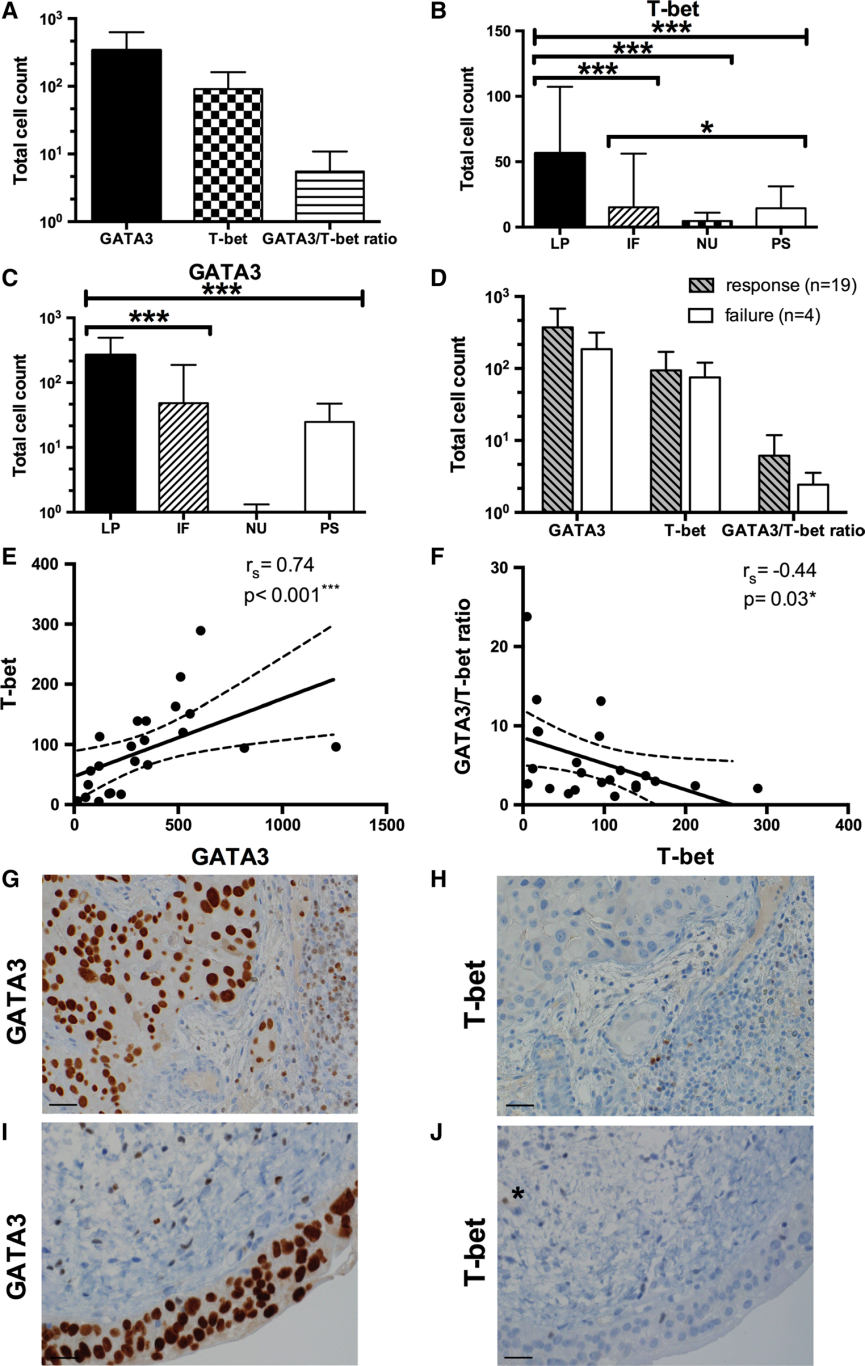
GATA3 and T-bet expression of tumor-infiltrating immune cells prior to BCG therapy. **a** Distribution of GATA3^+^, T-bet^+^ T cells and GATA3/T-bet ratio in all patients; data represent mean ± SEM. **b**, **c** Abundance of GATA3^+^ and T-bet^+^ T cells based on analyzed tumor areas. Data represent mean ± SEM. *p < 0.05; **p < 0.01; ***p < 0.001; Friedman’s tests and Wilcoxon’s signed-rank tests. **d** Frequency of GATA3^+^, T-bet^+^ tumor-infiltrating T cells and GATA3/T-bet ratio with respect to treatment outcome (response vs. failure). Data represent mean ± SEM; Mann–Whitney *U* test. **e**, **f** Correlation analysis (and confidence bands) of GATA3, T-bet and GATA3/T-bet ratio; *p < 0.05; **p < 0.01; ***p < 0.001. **g** Superficially invasive bladder cancer with a high count of GATA3^+^ tumor-infiltrating lymphocytes and **h** low expression of T-bet^+^ T cells; *Scale bar* ~40 µm; **i** carcinoma in situ with few GATA3^+^ T cells and **j** only one intravascular T-bet^+^ T cell (marked with *); *Scale bar* ~40 µm. *LP* lamina propria without invasion, *IF* invasive front, *NU* neoplastic urothelium, *PS* papillary stroma, *r*_*s*_ Spearman’s rank correlation coefficient

IHC analysis showed a Th2 predisposition of tumor-infiltrating immune cells in therapy-naive patients: The mean (±SD, range) number of GATA3^+^ or T-bet^+^ immune cells and the GATA3/T-bet ratio was 340.2 (±288, 55–1258), 90.8 (±71.4, 12–289) and 5.5 (±5.3, 1.1–23.8) in all patients, respectively (Fig. 2a). Moreover, a GATA3/T-bet ratio >2 was confirmed in 20 (86.9%) of 23 patients and in 18 (94.7%) of 19 responders. Significant differences in the localization pattern and the density of immune cells between the four tumor regions were confirmed for T-bet (*p* < 0.001) and GATA3 (*p* < 0.001). The highest infiltration of GATA3^+^ (266.9 ± 226.8) and T-bet^+^ (56.4 ± 50.9) T cells was measured in the lamina propria without invasion, followed by the invasive front (48.3 ± 139.8 for GATA3; 15.2 ± 41 for T-bet; *p* < 0.001), the papillary tumor stroma (24.7 ± 22.7 for GATA3; 14.5 ± 16.7 for T-bet) and finally within the neoplastic urothelium (0.3 ± 1.0 for GATA3; 4.7 ± 6.3 for T-bet, *p* < 0.001), Fig. 2b, c. By analyzing differences in the expression of GATA3^+^ and T-bet^+^ immune cells between BCG response and failure, we found no statistically significant differences concerning GATA3, T-bet and GATA3/T-bet ratio. Nevertheless, BCG responders pointed out a clear tendency toward increased GATA3^+^ T cell counts (response vs. failure; 372.6 ± 303.6 vs. 186 ± 130.1, *p* = 0.218) and increased GATA3/T-bet ratio (6.2 ± 5.7 vs. 2.4 ± 1.1, *p* = 0.081), while the mean (±SD) T-bet^+^ cell expression was similar (94.1 ± 76.2 vs. 75 ± 45.6, *p* = 0.785), Fig. 2d. In BCG failure, post-BCG tumor tissue analysis revealed no dynamic changes (mean ± SD) for GATA3 (199 ± 91.7), T-bet (64 ± 30.8) and GATA3/T-bet ratio (2.1 ± 1.8) compared to baseline. Significant positive correlations between GATA3 and T-bet (*r*_s_ = 0.74, *p* < 0.001) were identified, while T-bet correlated inversely with GATA3/T-bet ratio (*r*_s_ = −0.44, *p* = 0.03), Fig. 2e, f. Representative IHC images of two patients with a higher density of GATA3^+^ T cells compared to T-bet^+^ T cell count are shown in Fig. 2g–j.

### Responders displayed increased levels of Th1-related inflammatory metabolites and decreased concentrations of IL-10 compared to non-responders

**Fig. 3:**
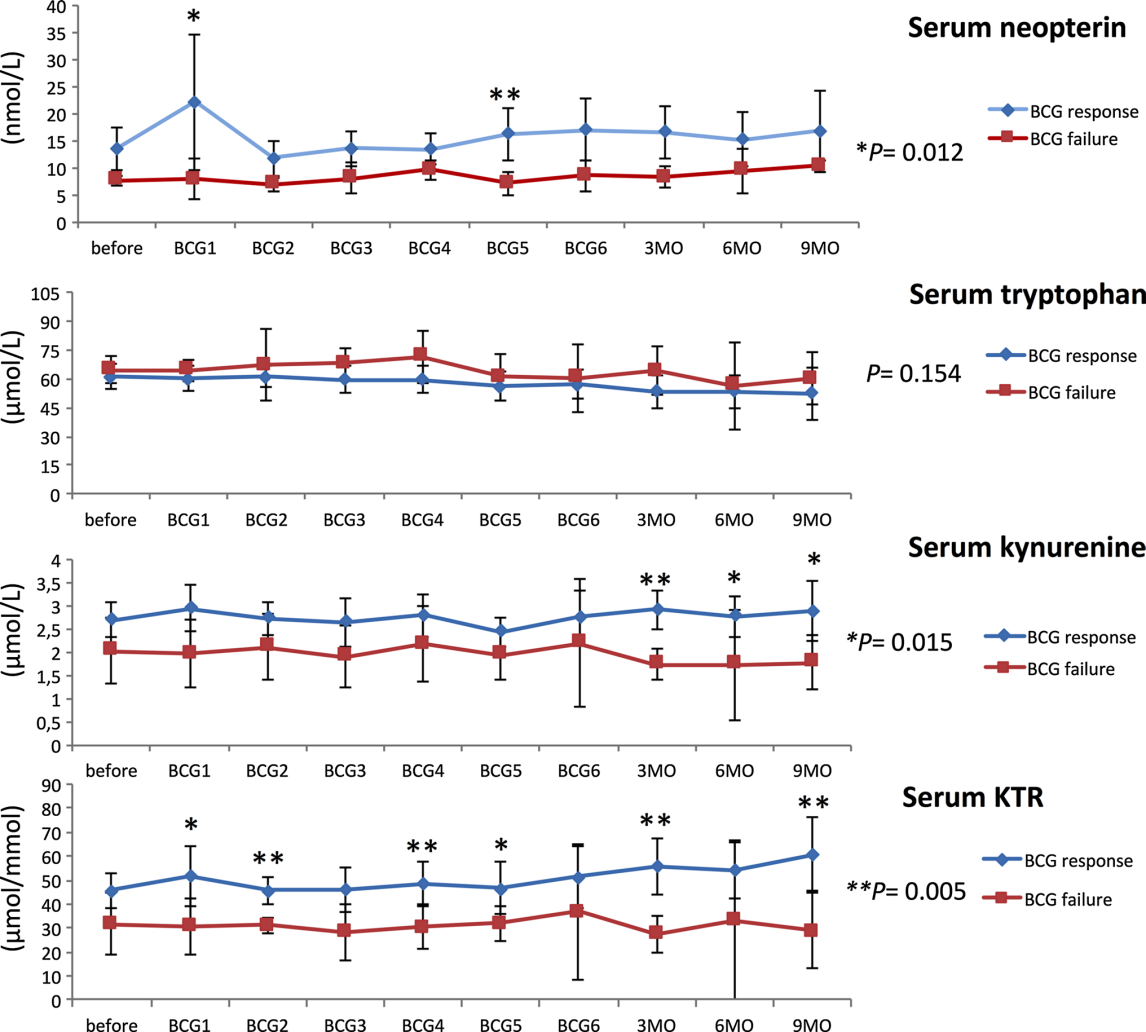
Levels of serum neopterin and tryptophan degradation at baseline, during and after BCG therapy. Patients were stratified by treatment outcome (BCG response vs. failure). Data represent mean ± 95% confidence interval (CI); *p < 0.05; **p < 0.01; ***p < 0.001; overall *p* value from a mixed model analysis for repeated measures between responders and non-responders; Mann–Whitney *U* tests for single time point comparisons. *KTR* kynurenine-to-tryptophan ratio

**Fig. 4:**
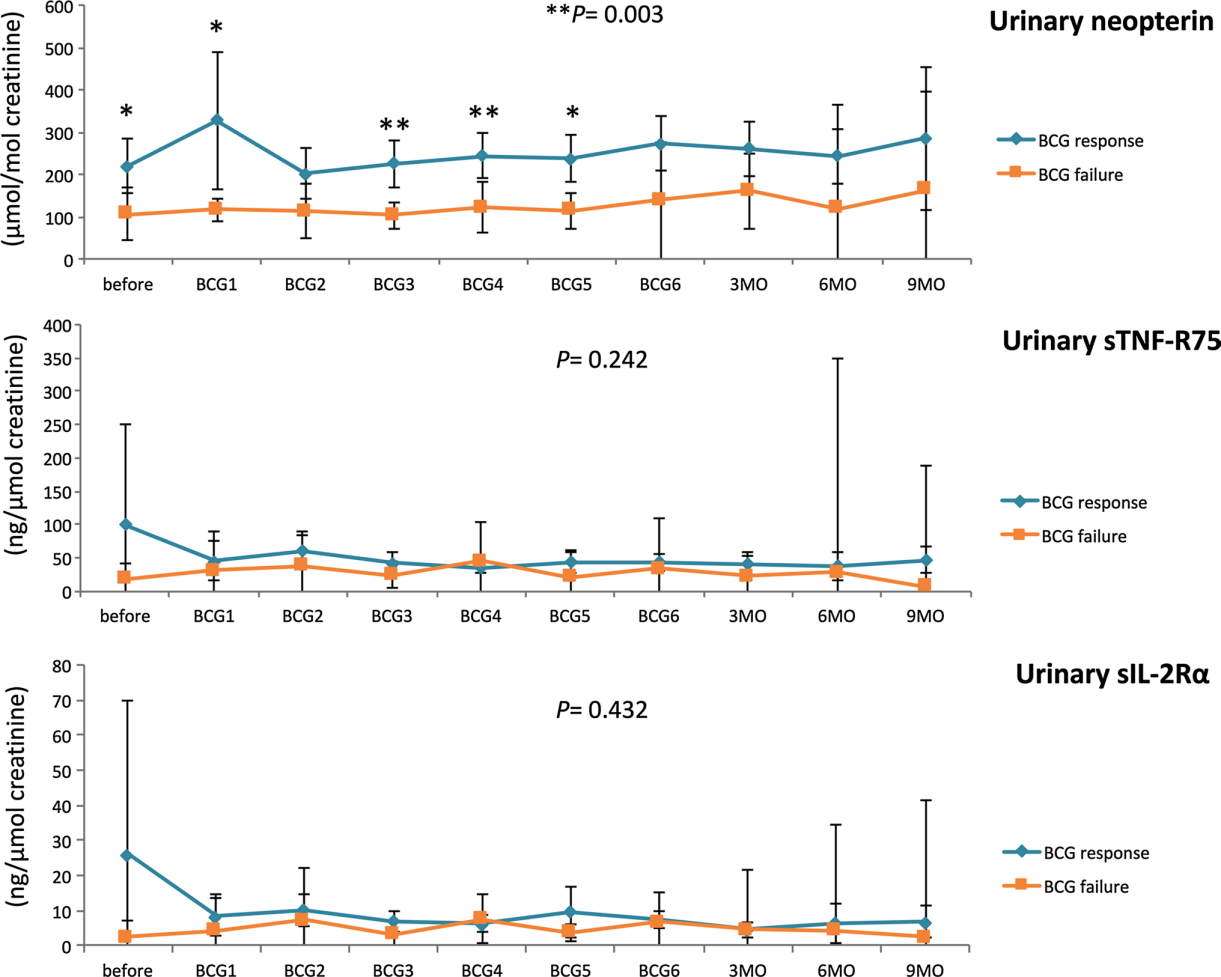
Levels of urinary neopterin, sTNF-R75 and sIL-2Rα (pro creatinine) at baseline, during and after BCG therapy. Patients were stratified by treatment outcome (BCG response vs. failure). Data represent mean ± 95% confidence interval (CI); *p < 0.05; **p < 0.01; ***p < 0.001; overall *p* value from a mixed model analysis for repeated measures between responders and non-responders; Mann–Whitney *U* tests for single time point comparisons. *s* soluble, *TNF* tumor necrosis factor, *IL* interleukin

**Fig. 5:**
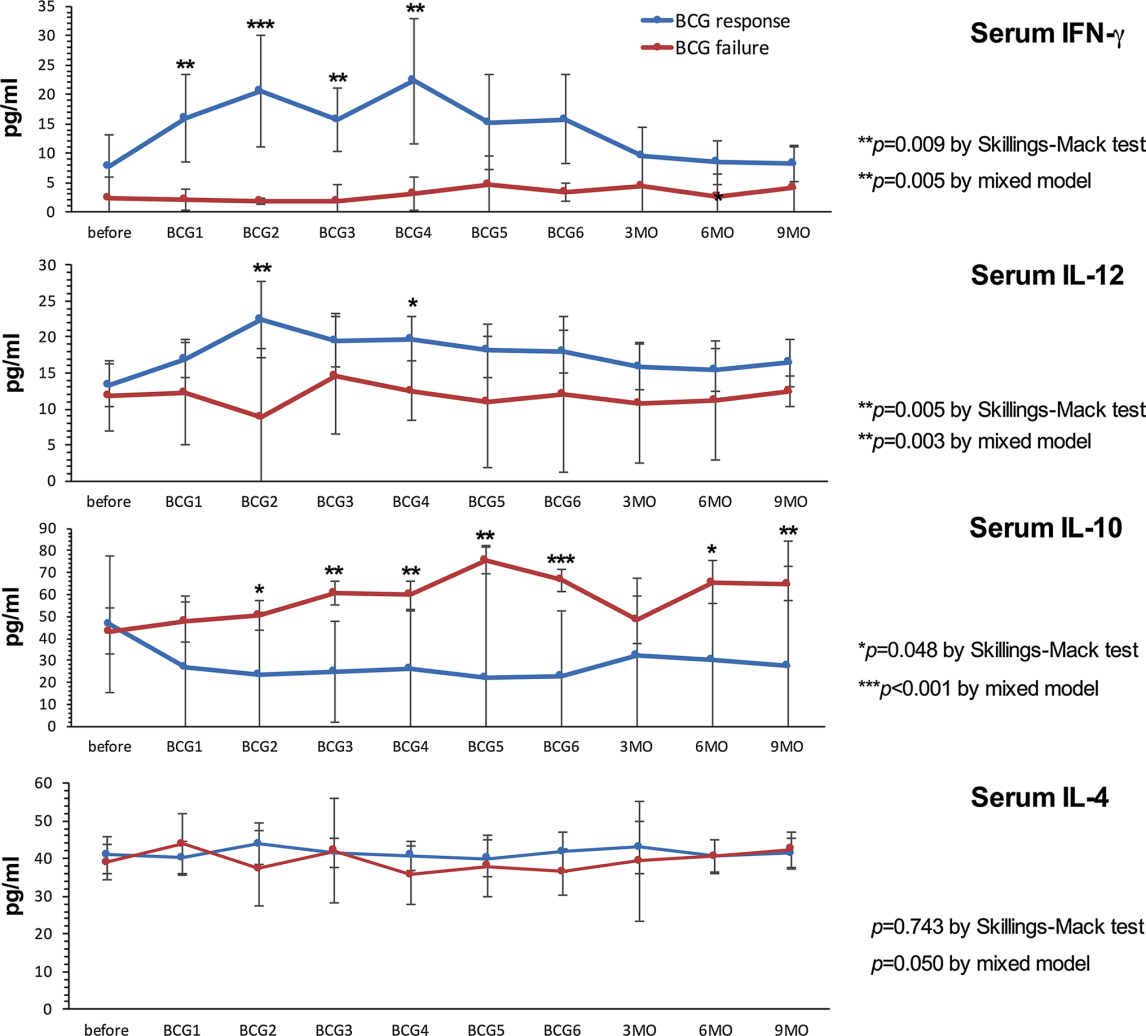
Levels of serum IFN-γ, IL-12, IL-10 and IL-4 (pg/ml) at baseline, during and after BCG therapy. Patients were stratified by treatment outcome (BCG response vs. BCG failure). Data represent mean ± 95% confidence interval (CI); *p < 0.05; **p < 0.01; ***p < 0.001; overall *p* value for changes over time points by Skillings–Mack test; *p* value from a mixed model analysis for repeated measures between responders and non-responders; Mann–Whitney *U* tests for single time point comparisons between responders and non-responders. *IL* interleukin

Response to BCG has previously been linked to an increase in Th1-related cytokines during treatment [21, 22]. Neopterin production and tryptophan degradation, measurable in serum, are strongly inducible by the Th1-type cytokine IFN-γ [38] and thus may reflect Th1 signals induced locally within the bladder by BCG. In our study population, we observed significant time-dependent changes for serum tryptophan (*p* = 0.024), IFN-γ (*p* = 0.009), IL-12 (*p* = 0.005) and IL-10 (*p* = 0.048), and a tendency toward an increase in serum (nmol/l; *p* = 0.056) and urinary (µmol/mol creatinine; *p* = 0.059) neopterin production during BCG therapy. Dichotomizing into patients with BCG response and failure, we noticed significantly higher increases over the course of time in BCG responders compared to non-responders, for serum neopterin (*p* = 0.012), serum kynurenine (μmol/l; *p* = 0.015), serum KTR (μmol/mmol; *p* = 0.005), urinary neopterin (*p* = 0.003), serum IFN-γ (pg/ml; *p* = 0.005) and IL-12 (pg/ml; *p* = 0.003) concentrations, whereas the levels of Th2 cytokine IL-10 (pg/ml; *p* < 0.001) were significantly lower in responders compared to non-responders (Figs. 3, 4, 5). At baseline, only urinary neopterin levels were significantly higher in BCG responders compared to BCG failure (mean ± SD, 219 ± 133 vs. 107 ± 38.2; *p* = 0.021), Fig. 4. ROC analysis showed that the best cutoff (=highest Youden Index: 0.789) for urinary neopterin was ≥137.5 μmol/mol creatinine, with 78.9% sensitivity and 100% specificity in the prediction of BCG response [area under the curve (AUC) = 0.862; 95% CI 0.707–1.00; *p* = 0.026].

With regard to changes from baseline to the time after the first BCG instillation, BCG responders could be identified by an increased level of serum neopterin (mean ± SD, 22.2 ± 25.9 vs. 7.9 ± 2.3; *p* = 0.044), serum KTR (mean ± SD, 51.8 ± 24.6 vs. 30.7 ± 7.2; *p* = 0.024), serum IFN-γ (mean ± SD, 15.9 ± 15.3 vs. 2.1 ± 1.1; *p* = 0.003) and urinary neopterin (mean ± SD, 328 ± 336 vs. 117 ± 17.6; *p* = 0.016). Dynamic changes in serum kynurenine concentrations were observed after completed BCG induction: BCG responders displayed a significant increase in 3 months (mean ± SD, 55.7 ± 19.9 vs. 27.6 ± 4.8; *p* = 0.001), 6 months (mean ± SD, 54.3 ± 19.2 vs. 33.1 ± 13.0; *p* = 0.014) and 9 months (mean ± SD, 60.9 ± 20.3 vs. 28.8 ± 6.4; *p* = 0.028) after BCG induction in comparison with BCG failure. In contrast, no significant differences of serum IL-4, urinary sIL-2Rα or sTNF-R75 levels could be noticed in response to BCG therapy, Figs. 3, 4 and 5.

### Significant correlations between urinary and serum Th1-type immune response-associated markers at baseline

As a next step, we investigated the extent to which urinary and serum neopterin production correlated with tryptophan breakdown. Correlation analyses revealed significant associations of serum neopterin with urinary neopterin (*r*_s_ = 0.761, *p* < 0.001), serum tryptophan (*r*_s_ = -0.395, *p* = 0.069), serum kynurenine (*r*_s_ = 0.783, *p* < 0.001), serum KTR (*r*_s_ = 0.817, *p* < 0.001) and serum IFN-γ (*r*_s_ = 0.462, *p* = 0.026). Moreover, urinary sTNF-R75 correlated significantly with urinary sIL-2Rα (*r*_s_ = 0.744, *p* < 0.001), Supplementary Figure 2.

#### Intratumoral GATA3/T-bet ratio correlated with serum neopterin, IFN-γ and KTR levels after first BCG instillation

Consistent with the hypothesis that a predominant Th2 tumor microenvironment relates to a functional Th1 response during BCG treatment, we confirmed a positive correlation between GATA3/T-bet ratio prior BCG induction and levels of serum neopterin (*r*_s_ = 0.533, *p* = 0.008), IFN-γ (*r*_s_ = 0.505, *p* = 0.013) and KTR (*r*_s_ = 0.508, *p* = 0.018) after the first BCG instillation, respectively.

### CD4 expression in Th cells increased significantly during BCG therapy

**Fig. 6:**
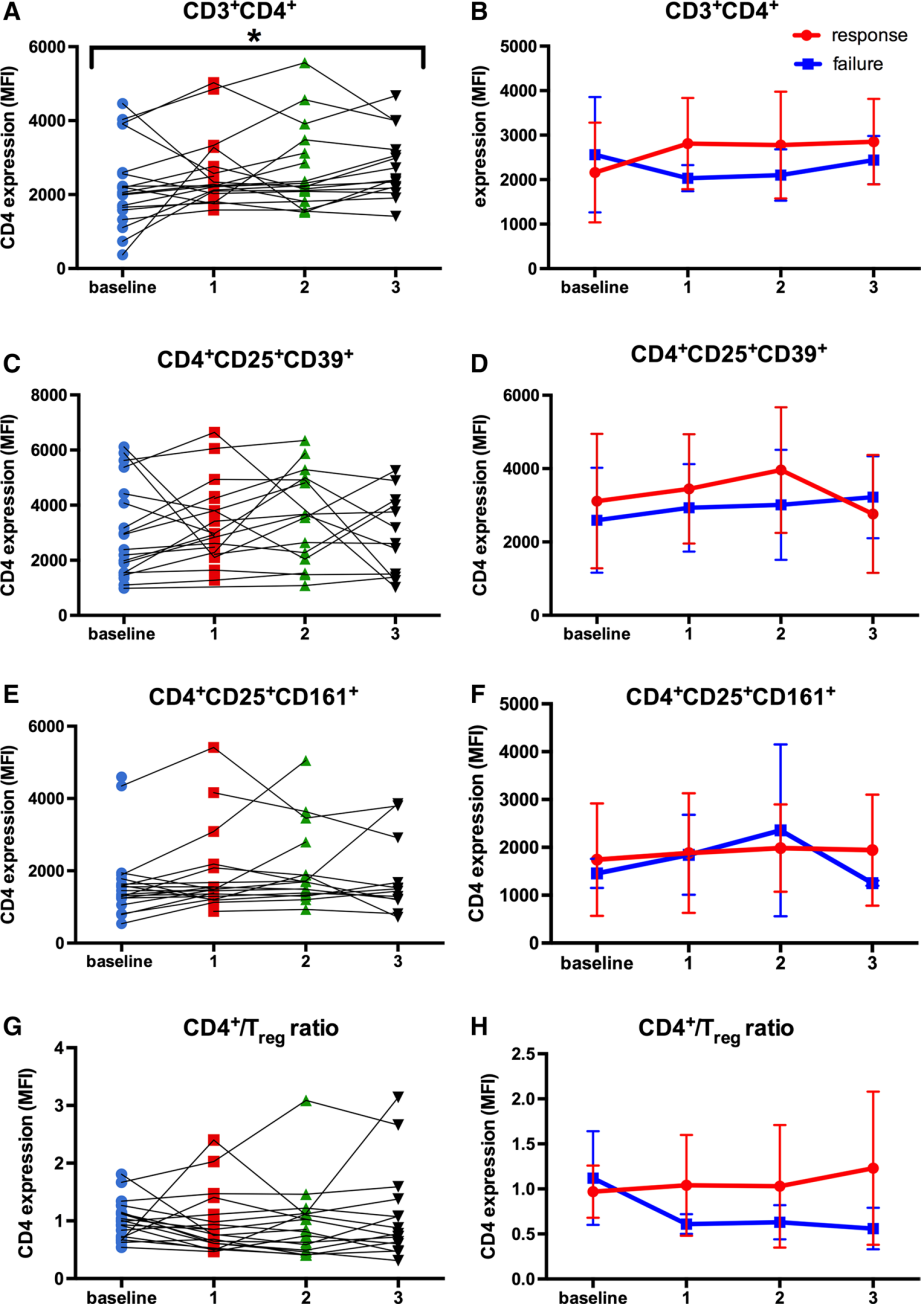
CD4 expression in the Th cell population, CD4^+^CD25^+^CD39^+^ T_reg_ cells and CD4^+^CD25^high^CD161^+^ T_eff_ cells, and CD4^+^/T_reg_ ratio. **a** Significant changes in the CD4 expression in Th cells at baseline and during treatment in all patients; Skillings–Mack test; scatter dot plot. **b** CD4 expression in Th cells at baseline and throughout treatment, depending on therapeutic outcome (response vs. failure); mixed model analysis for repeated measures and Mann–Whitney *U* tests for single time point comparisons. Data represent mean ± SD; **c** no significant dynamic changes concerning the CD4 expression in T_reg_ at baseline and during treatment in all patients; Skillings–Mack test; scatter dot plot. **d** CD4 expression in T_reg_ cells at baseline and throughout treatment, depending on therapeutic outcome (response vs. failure); mixed model analysis for repeated measures and Mann–Whitney *U* tests for single time point comparisons. Data represent mean ± SD; **e** no significant dynamic changes concerning the CD4 expression in T_eff_ cells at baseline and during treatment in all patients; Skillings–Mack test; scatter dot plot. **f** CD4 expression in T_eff_ cells at baseline and throughout treatment, depending on therapeutic outcome (response vs. failure); mixed model analysis for repeated measures and Mann–Whitney *U* tests for single time point comparisons. Data represent mean ± SD; **g** no significant dynamic changes concerning the CD4 expression in total CD4^+^/T_reg_ ratio at baseline and during treatment in all patients; Skillings-Mack test; Scatter dot plot. **h** CD4 expression in CD4^+^/T_reg_ ratio at baseline and throughout treatment, depending on therapeutic outcome (response vs. failure); mixed model analysis for repeated measures and Mann–Whitney *U* tests for single time point comparisons. Data represent mean ± SD; *p < 0.05; **p < 0.01; ***p < 0.001; *MFI* mean fluorescence intensity

CD3^+^ and CD4^+^ T cells as well as T_reg_ cells have shown to differentially influence BCG vaccine efficacy [39, 40], being potential candidates for monitoring response to BCG immunotherapy. As shown in Fig. 6a, the CD4 expression in Th cells changed significantly during BCG induction (*p* = 0.049). In detail, the CD4 expression in Th cells increased steadily from baseline (mean ± SD, 2144.3 ± 1132.8) to the third BCG instillation (2770.2 ± 891.8), respectively (Fig. 6a). Based on therapy outcome, BCG responders showed a tendency toward an increased CD4 expression in Th cells during treatment compared to BCG failure, already after the first BCG instillation (2686.1 ± 995.3 vs. 1931.3 ± 261.1; *p* = 0.109), but without statistical significance, Fig. 6b. In contrast, the CD4 expression in CD4^+^CD25^+^CD39^+^ T_reg_ and CD4^+^CD25^high^CD161^+^ T_eff_ cell populations remained relatively stable in the total population as well as stratified by therapy response (Fig. 6c–f). Moreover, we noticed no significant differences neither in the CD4^+^/T_reg_ ratio of the total study population (Fig. 6g) nor in the change of the CD4^+^/T_reg_ ratio during therapy between responders and non-responders (Fig. 6h). However, BCG responders showed a trend toward increased CD4^+^/T_reg_ ratio compared to BCG failure (after three instillations, 1.26 ± 0.85 vs. 0.69 ± 0.12, *p* = 0.571). In addition, we observed no significant correlations between the CD4 expression in Th cells, T_reg_ and T_eff_ cells, and the serum concentrations of neopterin and tryptophan degradation.

## Discussion

Immunity to pathogens including mycobacteria is mediated by type 1 effector Th cells (Th1 cells), which produce IFN-γ [27]. Whereas the T cell transcription factor T-bet (encoded by *Tbx21*) has a fundamental role in coordinating a type 1 immune response, GATA3 is considered to be the master regulator of the Th2 cell differentiation program characterized by the production of IL-4, IL-5 and IL-13. T-bet also promotes Th1-type responses by preventing GATA3-mediated Th2 cell development [41, 42]. Despite this established concept of mutually exclusive Th cell differentiation, there is also evidence that Th2-type cytokines such as IL-4 are important for memory T cell generation [43] and for the development of CD8^+^ T cell immunity against intracellular parasites [44].

A Th2 (GATA3>T-bet) predisposition has been reported in different cancer entities and metastatic lymph nodes and was found to be associated with cancer recurrence, progression and poor survival [45–48]. This fact may support the hypothesis of an inverse correlation of GATA3 and T-bet expression, suggesting that the presence of Th2 immune cells limits functional Th1-type responses within the tumor microenvironment. In the present study, we observed a positive correlation between GATA3 and T-bet expression in tumor-infiltrating immune cells. In detail, when GATA3 expression was high, also T-bet was increased and vice versa. Our present findings together with results recently published by our study group [30] clearly indicate a Th2 predisposition of tumor-infiltrating immune cells in BCG responders. However, increased levels of GATA3 expression and GATA3/T-bet ratio were significantly associated with a prolonged, and not a poor recurrence-free survival [30]. Moreover, our current data also showed the tendency toward increased numbers of GATA3^+^ immune cells in BCG responders compared to BCG failure. These results are comparable with the trial by Nunez-Nateras et al. [49] analyzing the pretreatment immunologic tumor microenvironment within tumor-infiltrating lymphocytes. They also showed an increased pretherapeutic GATA3/T-bet ratio in responders in comparison with BCG failure.

Using real-time PCR to asses the gene expression of GATA3 and T-bet, a reduced Th2 phenotype (decreased GATA3 expression) correlated with disease aggressiveness (high-grade tumors and muscle-invasive status) and a poor cancer-specific survival in bladder cancer [50]. In line with this observation, we observed that the Th2 cytokine IL-4 can directly suppress the growth of tumor cells [51]. Whereas GATA3 expression remained mostly negative in prostate and renal cell carcinoma, up to 70% of invasive urothelial carcinomas were GATA3 positive in one study [52]. However, real-time PCR analysis confirmed higher expression of T-bet among BCG responders as compared to BCG failure (*p* = 0.02), whereas heavy smokers with low expression levels of GATA3 were poor responders to BCG (*p* = 0.01), [50].

Focusing on tumor cells, GATA3 has been shown to prevent bladder cancer progression and metastasis by inhibiting cell migration and invasion as well as epithelial-to-mesenchymal transition in vitro [53]. On the contrary, strong expression of GATA3 was associated with progression and poor cancer-specific survival in muscle-invasive bladder tumors [54]. Thus, the role of GATA3 has to be further validated and needs to be interpreted with caution. A possible explanation of these conflicting results may be the interaction of GATA3 with steroid hormone receptor signals influencing bladder cancer growth adversely by both stimulatory and inhibitory effects via androgen receptor (AR), estrogen receptor (ER)α and/or ERβ [55, 56]. For example, GATA3 expression correlated with a loss of ERβ as well as with AR/ERα overexpression in bladder cancer [54].

It is well established that Th1-type immunity is required for effective BCG-induced antitumor activity in bladder cancer. BCG efficacy may be increased by ex vivo tumor antigen-loading and dendritic cell activation as BCG stimulated dendritic cells to secrete TNF-α, which is responsible for phenotypic and functional changes [57]. Ponticiello et al. [58], for instance, noticed a significant increase in the CD4^+^ Th1 subsets during BCG therapy. Moreover, BCG resulted in an increase in Th1 cytokines and lower levels of Th2 cytokines during therapy in vitro and in humans [13, 21, 22, 59, 60]. Moreover, parenteral exposure to BCG before instillations triggered an accelerated T cell entry into the bladder in vitro, with an improved recurrence-free survival in patients [60]. Therefore, shifting the Th2 dominant immunologic landscape toward a Th1 response during therapy seems to be an important mechanism for adequate BCG response [61]. Another likely point of view is that a certain Th2 capacity is necessary for the generation of an adequate Th1 response. For instance, exposure to the Th2 cytokine IL-4 has been shown to result in an enhanced CD8^+^ T cell response to pathogens, improving a proinflammatory Th1 immune response [43]. However, the exact mechanism how IL-4 alters the frequency of CD8^+^ T cells in humans is still unclear. In mice, IL-4-producing NKT cells correlated with thymic innate memory CD8^+^ T cells [62]. In renal cell carcinoma, IL-4 and TNF-α synergistically induced apoptosis and cytokine production in vitro, promoting the recruitment of different immune effector cells [51]. Thus, simultaneous induction of both Th1 and Th2 response seems to be necessary for efficient systemic antitumor activity [63, 64]. As an example, a Th2-biased response to MAGE-6 epitopes prior to treatment shifted to a Th1-mediated response after therapy in two patients (renal cell carcinoma and melanoma) with complete therapeutic response [65]. Our present results confirm this view, since patients with Th2 predominant tumor-infiltrating immune cells displayed a Th1 functional phenotype during BCG induction.

IFN-γ-induced neopterin production and tryptophan breakdown are typical markers for Th1-activated cell-mediated immunity [23, 24]. So far, only a letter to the editor assessed changes in neopterin levels during BCG. Mack et al. [25] showed for the first time in 30 patients that BCG response was associated with a significant peak of serum and urinary neopterin after each BCG instillation as a sign of a Th1 cell-mediated immune activation. Our results are in line with these data as BCG responders also displayed higher levels of urinary and serum neopterin during intravesical BCG induction. In addition, we demonstrate for the first time enhanced tryptophan breakdown in the serum of BCG responders. Moreover, concentrations of neopterin correlated significantly with those for tryptophan breakdown at each time point confirming that IFN-γ induces simultaneously induces two different biochemical pathways: first, deprivation of tryptophan by IDO1; second, neopterin and reactive oxygen species (ROS) production by GCH-I [66]. Moreover, it is remarkable that neopterin and tryptophan metabolites can be detected in human serum as surrogate markers of a local IFN-γ response occurring in the bladder or the draining lymph nodes after BCG therapy.

As a result of a chronically activated immune system, also counter-regulatory and immunosuppressive mechanisms can be activated with decreased T cell responsiveness and development of immunodeficiency as a consequence of T_reg_ cell expansion [67, 68]. A positive correlation was found between high IDO1 expression in bone marrow-derived mesenchymal stem cells and elevated percentage of T_reg_ cells in acute myeloid leukemia [69]. Therefore, the proportion of CD4^+^CD25^+^CD39^+^ T_reg_ cells was analyzed in PMBCs at baseline, during and after BCG therapy. However, we noticed no significant correlations between serum levels of neopterin or tryptophan degradation and distribution of T_reg_ cells in PBMCs. Nevertheless, BCG responders showed the tendency toward increased T_reg_ cells compared to BCG failure, but without statistical significance.

The obvious limitation of this prospective pilot study is the relatively small sample size of 23 patients, which restricts statistical methods of interpretation. Therefore, further prospective and multi-institutional randomized trials with sufficient statistical power and long-term follow-up are required to validate these preliminary findings and to verify in detail the prognostic role of Th1-related inflammatory metabolites in the context of Th2-driving transcription factor overexpression.

## Conclusions

In patients receiving intravesical BCG therapy, a general intratumoral Th2 predisposition at the level of transcription factors (GATA3>T-bet) was combined with an increased productive Th1-type immunity in responders compared to non-responders. A better understanding of Th1 regulation by Th2 components at the molecular level would be helpful in developing a more efficient and targeted cancer immunotherapy, particularly in bladder cancer patients with BCG failure.

### Electronic supplementary material

Below is the link to the electronic supplementary material.

Supplementary material 1 (PDF 210 kb)

## Abbreviations

AR: Androgen receptor
AUC: Area under the curve
BCG: Bacillus Calmette–Guérin
CAR: Chimeric antigen receptor
ER: Estrogen receptor
ELISA: Enzyme-linked immunosorbent assay
FACS: Fluorescence-activated cell sorting
GCH-I: GTP cyclohydrolase
HPLC: High-performance liquid chromatography
IDO: Indoleamine 2,3-dioxygenase
IFN: Interferon
IL: Interleukin
KTR: Kynurenine-to-tryptophan ratio
NKT: Natural killer T cell
NMIBC: Non-muscle-invasive bladder cancer
PBMC: Peripheral blood mononuclear cell
ROC: Receiver operating characteristic
ROS: Reactive oxygen species
Th: T helper
T_eff_: Effector T cell
T_reg_: Regulatory T cell
TNF: Tumor necrosis factor
TURB: Transurethral resection of the bladder
