# Supplementary material for: Intratumoral Th2 predisposition combines with an increased Th1 functional phenotype in clinical response to intravesical BCG in bladder cancer
Source: Cancer Immunol Immunother. 2016 Dec 22;66(4):427–40. doi: 10.1007/s00262-016-1945-z (PMC5359386; doi:10.1007/s00262-016-1945-z)
Supplement: Supplementary file 1 — Supplementary material 1 (PDF 210 kb) [file 262_2016_1945_MOESM1_ESM.pdf]

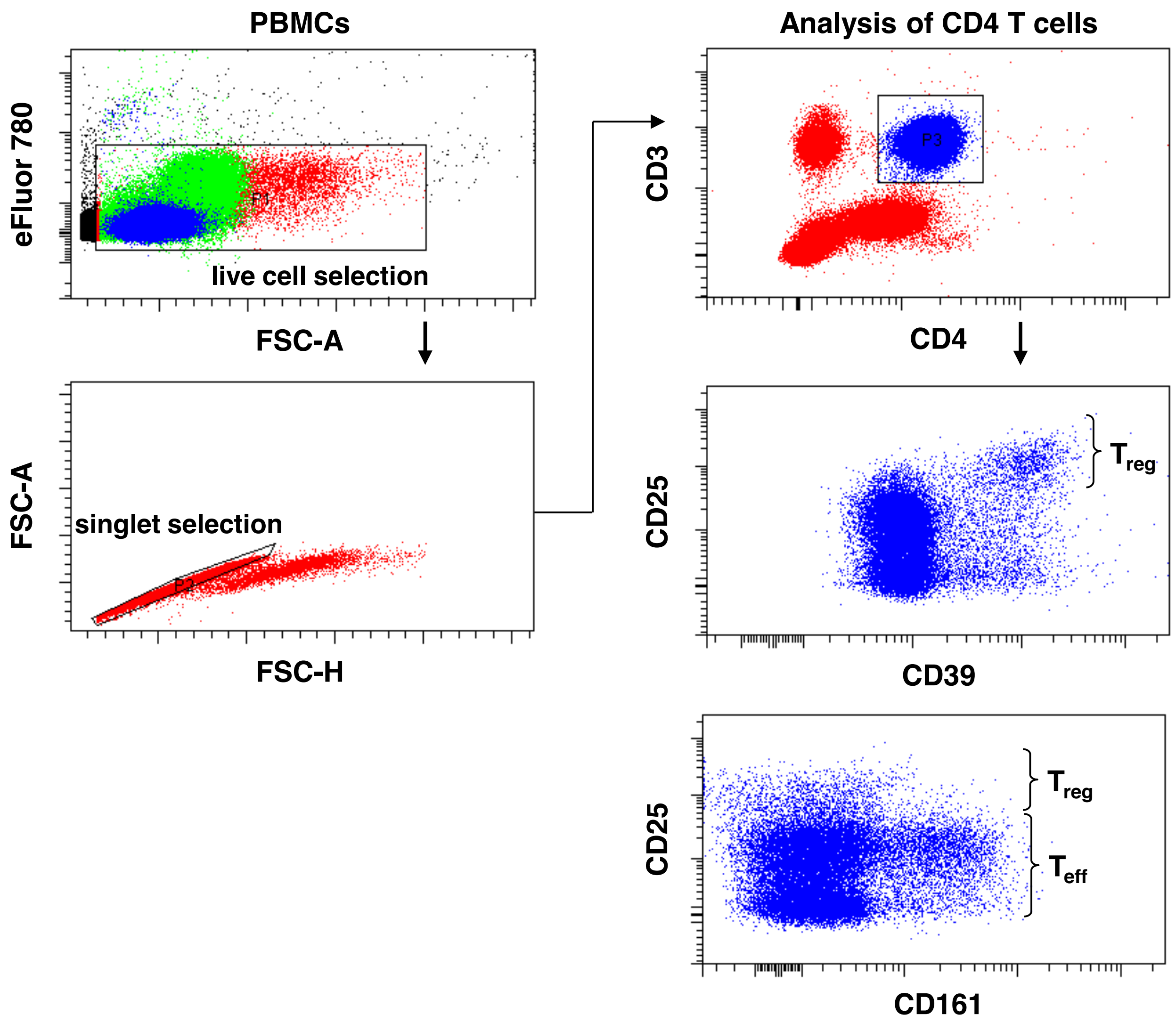

Supplementary Figure 1. Gating strategy for flow cytometry analysis. Live-dead discrimination and doublet discrimination by the indicated cell gating, analysis of $CD4^{+}$ T cells, $CD4^{+}CD25^{+}$ T cells, differentiation of effector T cells (T_{eff}) and regulatory T cells (T_{reg}) based on CD39 and CD161.

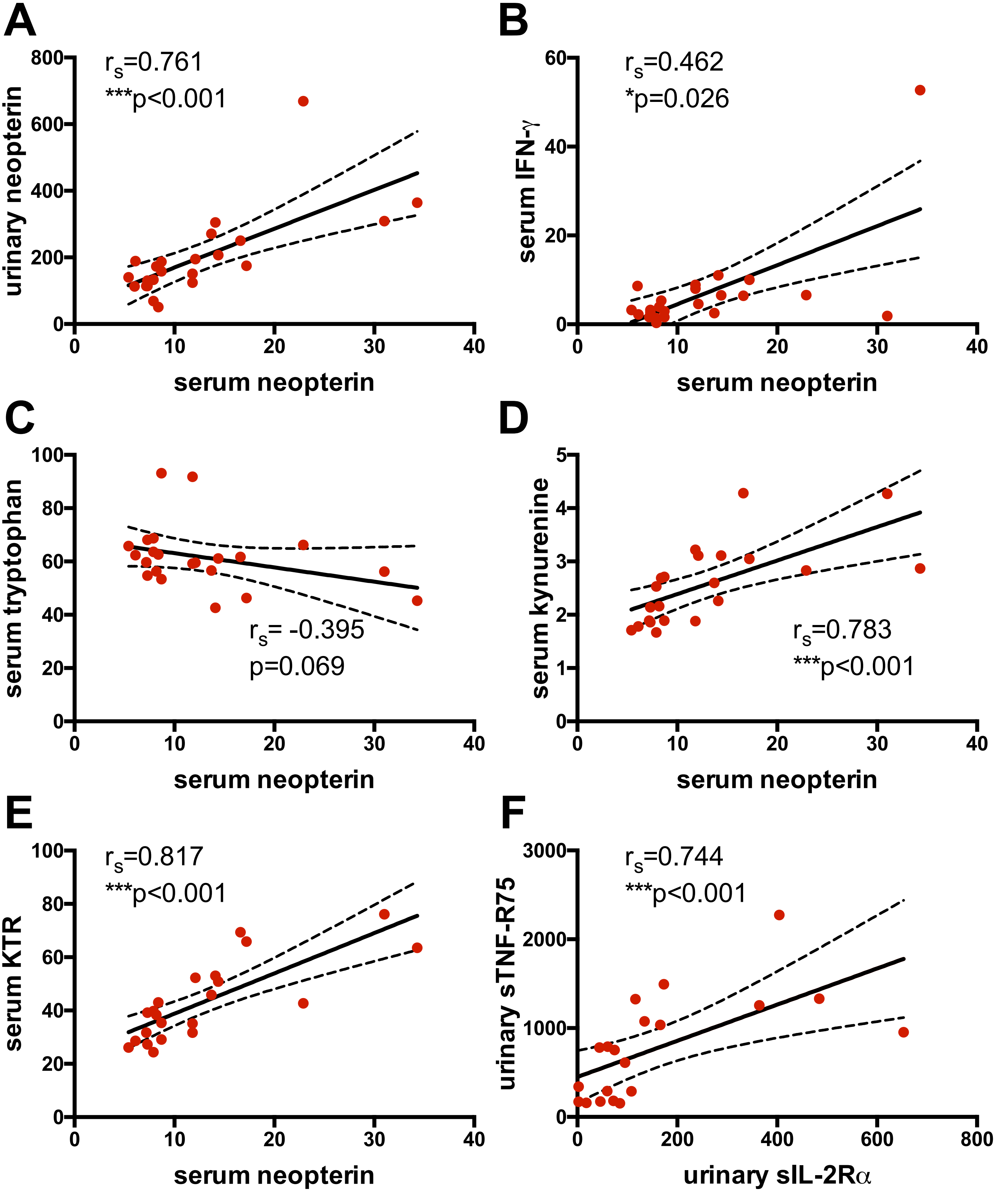

Supplementary Figure 2. Correlation analysis (and 95% confidence bands) at baseline between serum neopterin and A) urinary neopterin; B) serum IFN-γ; C) serum tryptophan; D) serum kynurenine; E) serum KTR; and F) urinary sIL-2Rα and sTNF-R75. P-values for r_s were corrected for multiple testing according to the Bonferroni method. *p<0.05; **p<0.01; ***p<0.001.

Abbrevations: Spearman`s rank correlation coefficient (r_s)

**Parameters**	**Response** (n=19)	**Failure** (n=4)
**Age**, mean (SD), *years*	71.8 (7.1)	68.7 (9.5)
**Gender,** n (%)		
Male	16 (84.2%)	4 (100%)
Female	3 (15.8%)	-
**Tumor diameter**, n (%)		
< 3 cm	11 (57.9%)	3 (75.0%)
≥ 3 cm	8 (42.1%)	1 (25.0%)
**Number of tumors**, n (%)		
Single	8 (42.1%)	-
2-7	7 (36.8%)	2 (50.0%)
>7	4 (21.1%)	2 (50.0%)
**T stage**		
pTa	9 (47.4%)	-
pT1	7 (36.8%)	2 (50.0%)
pTa + pT1	1 (5.3%)	-
CIS	2 (10.5%)	2 (50.0%)
**Concurrent CIS**	1 (5.3%)	2 (50.0%)
**Second TURB**, n (%)	16 (84.2%)	3 (75.0%)

Supplementary Table 1. Descriptive and histopathological characteristics based on oncological outcome to BCG.
